# Biochemical, serological, and genetic aspects related to gene HLA‐DQB1 and its association with type 1 diabetes mellitus (T1DM)

**DOI:** 10.1002/mgg3.1147

**Published:** 2020-03-06

**Authors:** Gur Charn Singh, Mehboob Ahmed, Muhammad Zaid, Shahida Hasnain

**Affiliations:** ^1^ Department of Microbiology and Molecular Genetics University of the Punjab Lahore Pakistan; ^2^ Department of Life Sciences School of Science University of Management and Technology Lahore Pakistan

**Keywords:** DQA1, DQB1, DRB1, HLA gene, hyperglycemia, type 1 diabetes mellitus (T1DM)

## Abstract

**Background:**

Type 1 Diabetes Mellitus (T1DM) is the autoimmune disorder of destruction of β cells of pancreas, creating insulin deficiency condition, which leads to hyperglycemia, polyuria, polydipsia, ketoacidosis, and other metabolic disorder especially in children. Different genetic aspects and environmental factors are involved in pathophysiology of the disease. About 20 genes are associated with this disease in which the most common is the different combination of haplotype DRB1‐DQA1‐DQB1 present at HLA gene. At HLA‐DQB1, there are some SNPs which are associated with T1DM. In T1DM, there are number of biochemical, serological parameters which show some abnormalities leading to some complications.

**Methods:**

Samples were subjected to all biochemical and serological techniques to get the measurement of concentration of glucose, lipid profile (cholesterol, triglycerides, and HDL and LDL cholesterol), urea, creatinine, albumin, insulin, anti‐insulin antibodies, C‐peptides, and leptin. All these values were compared with controls values and statistical analysis was also done on these values. At molecular level, two primers set which were allele specific at HLA‐DQB1, were used to amplify the SNPs, homozygous and heterozygous conditions were stated.

**Results:**

PCR results for the studied population showed that most of samples have heterozygous condition for these SNPs of this allele specific region on HLA‐DQB1. Very few of them have homozygous state for it. Even in the control sample have the same conditions.

**Conclusion:**

In Pakistan, there is dire need of studies about SNPs and haplotypes related to HLA‐DQB1 which show association with T1DM.

## Graphical abstract

Different genetic aspects and environmental factors are involved in the pathophysiology of Type1 diabetes mellitus. About 20 genes are associated with this disease in which the most common is the different combination of haplotype DRB1‐DQA1‐DQB1 present at HLA gene. At HLA‐DQB1, there are some SNPs which are associated with T1DM

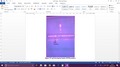

## 1 INTRODUCTION

The term diabetes mellitus describes a metabolic disorder of multiple etiology, characterized by chronic hyperglycemia with disturbances of carbohydrate, fat, and protein metabolism resulting from defects in insulin secretion, insulin action, or both. Type 1 diabetes (T1D) is a multifactorial autoimmune disease characterized by insulin deficiency, due to the T‐cell‐mediated destruction of pancreatic b cells (Al‐Mutairi, Mohsen, & Al‐Mazidi, 2007; Katsarou et al., 2017). The rapidly progressive form of beta‐cell destruction is commonly observed in children having diabetes mellitus, but also may occur in adults referred as latent autoimmune diabetes in adults (LADA) (American Diabetes Association, 2014; Katsarou et al., 2017). The main genetic risk for T1D development is due to HLA class II molecule, particularly DR and DQ region, due to which autoantibodies formation cause the destruction of β cell of pancreatic cell populations (Pociot & Lernmark, 2016). There are 61 different chromosomal loci, which have been linked to type 1 diabetes (T1D) susceptibility in human's populations. Fifty percent of the genetic risks for T1D development is due to HLAs Class, leading to altered immune genes. Other non‐HLAs genetic variants for T1D susceptibility are poorly understood (Nyaga, Vickers, Jefferies, Perry, & O'Sullivan, 2018; Ounissi‐Benkalha & Polychronakos, 2008; Ram et al., 2016). The HLA region is a cluster of genes located within the major histocompatibility complex (MHC) on chromosome 6p21 having 4Mbp box. Altered molecules could be a genetic variant for the production of large number of autoimmune diseases and infections (Matzaraki, Kumar, Wijmenga, & Zhernakova, 2017). It is clear that some combinations of HLA‐DQ genes are associated with susceptibility to T1D. From both human genetics and animal model studies there is good evidence that particular alleles of the haplotype of HLA‐DQA1, DQB1, and DRB1 loci all are primarily involved in the genetic predisposition to T1D (Parkkola et al., 2017). There is secretion of some hormone and antibodies, which shows association with T1DM. These are insulin, anti‐insulin, C‐peptide, and leptin. Insulin is a polypeptide hormone that regulates carbohydrate metabolism. There are several conditions in which insulin disturbance is pathologic: for example, diabetes mellitus (type 1 diabetes mellitus and type 2 diabetes mellitus), insulinoma, metabolic syndrome, and polycystic ovary syndrome (Ando et al., 2008; Bonifacio et al., 2015). Anti‐insulin antibodies are secreted in serum during type 1 DM. Numerous researchers described the appearance of antibodies directed against the islet cells and insulin as the causal reason for the onset of the disease (Sahin, Cetinkalp, Ozgen, Saygili, & Yilmaz, 2010; Segal et al., 2008). C‐peptide of insulin is the C‐terminal cleavage product produced during processing of the insulin pro‐hormone to the mature insulin molecule. The pancreas of patients with type 1 diabetes is unable to produce insulin and they will therefore usually have a decreased level of C‐peptide (Jones & Hattersley, 2013). Leptin is a protein hormone with important effects in regulating body weight, metabolism, and reproductive function. There are studies that shows that serum leptin level is increased in type 1 diabetes mellitus endothelial (Iacobellis, Diaz, Mendez, & Goldberg, 2014; Kraus, Herman, & Kahn, 2010).

## 2 MATERIALS AND METHODS

### 2.1 Sampling

Sampling was divided in two sessions, control sampling and patient sampling. The age range was 6–16th year. All children who were selected for blood sampling were totally energetic and active. No one was lethargic. For control sampling, all normal children were selected. So for this, samples were obtained from two schools, Hira Public Model High School and Guru Nanak Ji Public Model High School, Nankana Sahib, Punjab, Pakistan. Patients samples were collected from, diabetic clinic in Children Hospital, Lahore. Prior to sample collection an informed consent was obtained from the parents of each study subject. The parents of the participants were required to sign the consent forms. However, the parents with no formal education were given the option to leave a thumb impression. To get serum, clotted vial was centrifuged at 6,000 rpm for 10–15 min and the serum samples were subjected to biochemical test. These serum vials were stored at −4°C to 4°C. The study protocols for the presented work were approved by the ethics committee of School of Biological Sciences, University of the Punjab, Lahore.

### 2.2 Biochemical analysis and ELISA

Concentration of glucose, total cholesterol, triglycerides, HDL, LDL, urea, creatinine, albumin, and HBA1c was determined in blood samples. All these tests were performed using commercially available kits (Spectrum). All ELISA testing was conducted on microwell plates and tests were performed using commercially available kits to determine the concentration of Insulin (Nova Tec), Anti‐Insulin (Nova Tec), C‐peptide (Nova Tec), and Leptin (LDN) in standards, samples, and controls.

### 2.3 Molecular techniques

#### 2.3.1 Isolation of DNA

Genomic DNA was extracted using peripheral leukocytes of whole blood by using Wizard® Genomic DNA purification kit, Promega. The tube of blood was gently rocked until thoroughly mixed. The extracted DNA was then stored at −20°C for further use. DNA was analyzed on 1% agarose gel.

#### 2.3.2 Polymerase chain reaction

For the optimization of annealing temperature, the gradient PCR with the temperature range from 51.5°C to 58.0°C was used. The maximum yield was at 54.3°C for first set primers and at 52.2°C for second set primers.

## 3 RESULT

In total, 15 patient samples and 10 control samples were collected. All of them were children of age range was 6–16 years. All collected data were arranged in tabular form in Table 1 for patient and Table 2 for controls.

**Table 1:** Different biochemical parameters and their concentration in patients samples

Subject	Age (year)	Gender	Weight (kg)	Height (cm)	Diabetes diagnosed since	Glucose (mg/dl)	Cholesterol (mg/dl)	Triglycerides (mg/dl)	HDL (mg/dl)	LDL (mg/dl)	Urea (mg/dl)	Creatinine (mg/dl)	Albumin (mg/dl)	HBA1c (%)	Diabetes in familial history	Exogenous insulin injection
P01	14	F	52	155	4 years	257	196	100	59	86	31	0.9	4.6	14.6	Grandfather (paternal)	Insulin N and insulin R
P02	12	M	35	152	2 years	436	145	111	64	102	36	1.0	3.9	9.7	None	Insulin N and insulin R
P03	12	F	42	143	5 years	282	148	146	58	84	37	0.7	4.1	8.6	Elder brother and father's sister	Insulin 70/30
P04	8	M	25	125	6 years	379	83	93	72	88	26	0.7	4.9	8.8	Grand father	Insulin 70/30
P05	8	F	23.5	147.5	8 months	154	113	78	69	78	33	0.8	4.5	8.1	None	Insulin N and insulin R
P06	16	M	53	172.4	3 years	215	163	78	56	88	32	0.7	4.4	8.6	Father and father's sister	Insulin NPH and insulin R
P07	11	M	33	140	1_1/2_ years	102	133	49	58	66	18	0.8	4.1	6.5	Grandmother, grandfather uncle (paternal) and mother	Insulin 70/30
P08	15	M	42	—	3 years	158	131	61	62	73	31	0.9	4.8	12.1	Grandmother (paternal)	Insulin N and insulin R
P09	11	F	27	—	3 years	120	149	72	68	68	18	0.9	3.9	8.3	Uncle (paternal)	Insulin NPH and insulin R
P10	9	M	24	—	4 years	170	114	72	65	94	18	0.7	4.3	9.2	Grandmother (paternal)	Insulin NPH and insulin R
P11	9	F	24	137	2 years	105	118	67	64	81	29	0.9	4.0	9.8	Grandmother (paternal)	Insulin N and insulin R
P12	9	F	28	123.7	2 months	69	141	72	65	107	36	1.0	4.2	8.2	Grandmother grandfather and uncle (maternal)	Insulin N and insulin R
P13	16	M	46.5	169	7 months	202	135	85	57	59	23	0.8	4.0	12.1	None	Insulin 70/30
P14	9	M	25	—	3_1/2_ years	151	95	112	69	71	32	1.2	3.9	8.2	None	Insulin NPH and insulin R
P15	11	M	28	136	2 years	303	118	69	71	71	18	1.0	4.0	8.1	Uncle	Insulin N and insulin R
Mean ± *SD*	11.33 ± 2.69	—	33.86 ± 10.21	145.50 ± 15.09	—	206.86 ± 102.37	132.13 ± 26.72	84.33 ± 23.75	63.8 ± 5.12	81.06 ± 13.08	27.86 ± 6.89	0.86 ± 0.13	4.24 ± 0.32	9.39 ± 1.99	—	—
Median	11	—	28	143	—	107	133	78	64	81	31	0.9	4.1	8.6	—	—
Variance	7.28	—	104.28	227.89	—	11,229.4	764.9	604.6	28.17	183.49	50.98	0.02	0.11	4.24	—	—
Minimum	8	—	23.5	123.7	—	69	83	49	56	59	18	0.7	3.9	6.5	—	—
Maximum	16	—	53	172.4	—	436	196	146	72	107	37	1.2	4.9	14.6	—	—
Range	8	—	29.5	48.7	—	367	113	97	16	48	19	0.5	1	8.1	—	—

**Table 2:** Different biochemical parameters and their concentration in control samples

Subject Identity	Age (year)	Gender	Weight (kg)	Height (cm)	Glucose (mg/dl)	Cholesterol (mg/dl)	Triglycerides (mg/dl)	HDL (mg/dl)	LDL (mg/dl)	Urea (mg/dl)	Creatinine (mg/dl)	Albumin (mg/dl)	HBA1c (%)
C01	14	M	44	150	93	154	95	52	60	22	0.7	4.6	5.0
C02	8	M	26	142	101	146	68	70	70	18	0.5	5.0	6.5
C03	11	M	35	132	83	135	105	58	72	29	0.9	4.6	6.7
C04	11	M	31	139	103	145	65	61	75	14	1.0	4.1	6.2
C05	13	M	38	149	102	150	61	69	63	18	1.0	4.4	7.2
C06	11	M	37	135	92	133	71	58	69	21	0.8	4.3	4.5
C07	14	M	46	142	82	102	92	62	65	31	0.7	4.6	6.0
C08	14	M	49	160	101	109	101	59	58	23	1.1	4.5	7.2
C09	14	M	46	152	78	134	78	62	65	13	0.7	4.5	6.8
C10	16	M	52	160	86	102	93	62	80	21	0.6	4.5	6.5
Mean ± *SD*	12.6 ± 2.2	—	40.4 ± 7.91	146.1 ± 9.22	92.1 ± 8.92	131 ± 18.72	82.9 ± 15.28	61.3 ± 5.00	67.7 ± 6.48	21 ± 5.47	0.8 ± 0.18	4.51 ± 0.22	6.26 ± 0.844
Median	13.5	—	41	145.5	92.5	134.5	85	61.5	67	21	0.75	4.5	6.5
Variance	4.84	—	62.64	85.09	79.69	350.6	233.49	25.01	42.01	30	0.034	0.04	0.71
Minimum	8	—	26	132	78	61	61	52	58	13	0.5	4.1	4.5
Maximum	16	—	52	160	103	105	105	70	80	31	1.1	5	7.2
Range	8	—	26	28	25	44	44	18	22	18	0.6	0.9	2.7

### 3.1 Biochemical reporting

The concentrations of different biochemical parameters were measured and these values present in Table 1 for patient and in Table 2 for controls. The highest value of glucose among patients was 436 mg/dl and in controls 103 mg/dl, which is in the normal range. The mean value of all patients is 206.86 mg/dl which is very high in comparison with controls samples, having mean value 92.1 mg/dl (Tables 1, 2). The results of cholesterol, triglycerides, HDL, and creatinine generally showed no abnormal values in control and also in patients (Tables 1, 2). In patient the LDL and urea content were at upper limit of the normal range as compare to controls value (Tables 1, 2). The mean value for Albumin in patients is lower in normal range as compared to the controls (Tables 1, 2). The Glycosylated hemoglobin (HBA1c) values were very high in patients. These patients were not in controlled situation for sugar level. Its mean value for patients is 9.33%, which shows that the sugar is in fair control in patients' blood (Table 1).

### 3.2 ELISA

After biochemical testing, ELISA was done to measure the concentration of insulin, anti‐insulin, c‐peptide, and leptin for patient and control samples. In all patients the insulin level are abnormally decreased 2.33 µlU/ml (Table 3), this concentration was much lower as compared to the control patients 5.89 µlU/ml as shown in Table 4. Some patients have fair level of insulin but this insulin may be the exogenous which are injected to the patients. The patients contain abnormally high level of anti‐insulin antibodies as compared to the control individuals. The mean value of patient anti‐insulin Ab is 13.93 U/ml which is almost double to the control mean value (6.01 U/ml). The values of c‐peptide are very low among patient if comparison is done with the control patients. Some patients do not contain c‐peptide. The control samples show mean value in normal range (0.95 ng/ml). As all lipids were in normal ranges that is why leptin concentration shows normality for all patients as well as controls (Tables 3, 4).

**Table 3:** Different parameters of ELISA and their concentration in patient samples

Subject	Age (year)	Gender	Insulin level µlU/ml	Anti‐insulin level U/ml	C‐peptide level ng/ml	Leptin level ng/ml
P01	14	F	0.3	17.5	0.2	8.7
P02	12	M	0.5	11.3	0.2	0.9
P03	12	F	1.3	14	0.1	8.9
P04	8	M	3.2	20.1	0.1	1.7
P05	8	F	5.3	21.5	0.0	1.6
P06	16	M	3.5	4.9	0.07	0.9
P07	11	M	4.2	14.1	0.05	4.9
P08	15	M	0.4	4.2	0.0	0.8
P09	11	F	5.8	23.3	0.02	1.8
P10	9	M	4.1	9.5	0.05	1.7
P11	9	F	4.1	37	0.02	1.1
P12	9	F	0.8	10.2	0.02	9.8
P13	16	M	0.8	3.5	0.025	0.8
P14	9	M	0.4	3.5	0.4	1.0
P15	11	M	0.3	14.4	0.01	4.1
Mean ± *SD*	11.33	—	2.33	13.93	0.084	3.24
Median	11	—	1.3	14	0.05	1.7
Variance	7.28	—	3.83	77.50	0.011	10.02
Minimum	8	—	0.3	3.5	0	0.8
Maximum	16	—	5.8	37	0.4	9.8
Range	8	—	5.5	33.5	0.4	9

**Table 4:** Different parameters of ELISA and their concentration in controls samples

Subject	Age (year)	Gender	Insulin level µlU/ml	Anti‐insulin level U/ml	C‐peptide level ng/ml	Leptin level ng/ml
C01	14	M	9.0	6	1.0	3.5
C02	8	M	9.6	4.8	0.8	8.7
C03	11	M	2.3	9.8	0.8	1.0
C04	11	M	3.3	8.0	0.7	1.9
C05	13	M	9.9	3	0.9	1.1
C06	11	M	5.0	9.0	1.3	2.1
C07	14	M	8.2	8.0	0.5	6.5
C08	14	M	3.0	3.0	1.2	3.4
C09	14	M	4.6	3.0	1.3	2.0
C10	16	M	4.0	5.5	1.0	1.8
Mean	12.6	—	5.89	6.01	0.95	3.2
Median	13.5	—	4.8	5.75	0.95	2.05
Variance	4.84	—	7.88	6.01	0.062	5.68
Minimum	8	—	2.3	3	0.5	1
Maximum	16	—	9.9	9.8	1.3	8.7
Range	8	—	7.6	6.8	0.8	7.7

### 3.3 Results of amplification of specific SNPs at HLA‐DQB1

**Figure 1:**
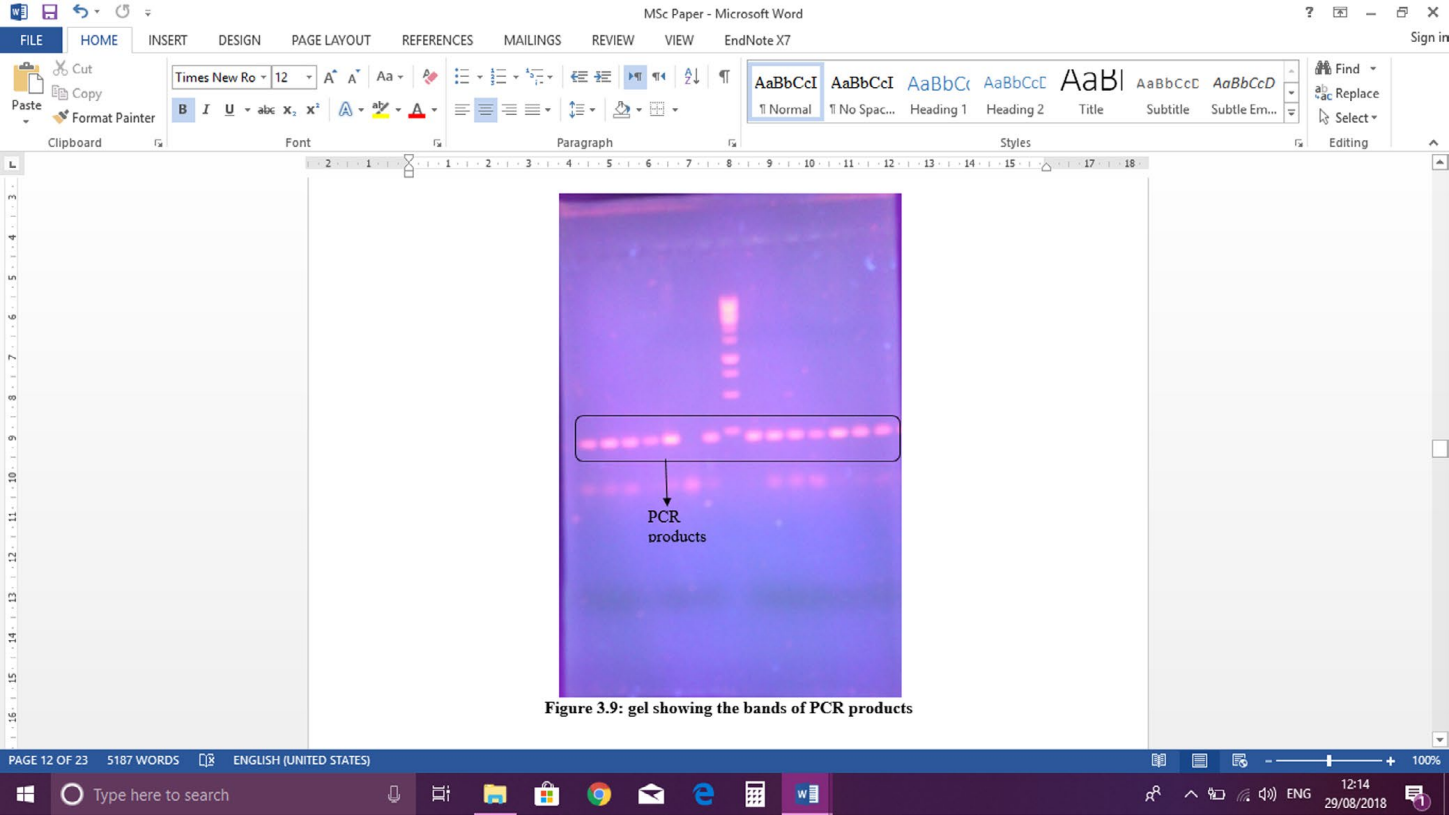
Gel showing the bands of PCR products

According to analysis of the type 1 diabetes genetics consortium families (Erlich et al., 2008), there are total 44 haplotypes which contain different combination of HLA‐DRB1‐DQA1‐DQB1. At HLA‐DQB1 there are number of SNPs in human population. PCR results (Figure 1) for the studied population showed that most of samples have heterozygous condition for these SNPs of this allele specific region on HLA‐DQB1. Very few of them have homozygous state for it. Even in the control sample have the same conditions.

## 4 DISCUSSION

About 2% of the diabetes patients are linked to type 1 diabetes mellitus. The age group for T1DM is 6–16 years, at which they are diagnosed. Mostly in very early ages, these patients are misdiagnosed with disease like stomach problems. There are number of complications in the course of T1DM. These complications may give the idea of stages of T1DM. The complications which may arise from diabetes include disease of the blood vessels, stroke, nerve damage, amputations, kidney failure, blindness, and foot fungous or gangrene. These complications reduce the life expectancy. There are numbers of prediction and indicators, which may tell about disease assessment for future days. Like routinely glucose estimation is first step for disease treatment. These patients require exogenous insulin for normal life to live. The doses of insulin directly affect the patient's health. These doses depend on the blood glucose level. Mainly, the glucose levels in these patients are at very higher level. The reason behind this, that these age groups (6–16) are related to schools periods, sports involvement, attending the parties. Therefore, these patients are more likely prone to hyperglycemia (higher glucose level condition) due to imbalance in diet. Glucose level may tell what would be the way to lowers the glucose level in body by insulin. These patients require attention of their parents, as they are closed to them in this period. The mean level glucose of those patients is 206 mg/dl. If it is well controlled by insulin doses, the glucose level may fall to <69 mg/dl. The highest level which is seen among subject (patients) is 436 mg/dl. Glucose concentration is the major concern for controlling the glucose level. The next indicator is the estimation of the concentration of lipid profile. Lipid profile includes cholesterol, triglycerides, HDL, and LDL cholesterol. The testing of these tests gives the information about patient that how much the patient is susceptible to get coronary and heart diseases. If these patients contain higher levels of some of lipid fractions, they may be on the stage to get heart related diseases which reduces the good health for normal life. The subjects (patient) which were taken under this project had normal lipid profile concentration (cholesterol, triglycerides, HDL, and LDL concentration). It means that these patients are on control way. Urea and creatinine are the next parameter that tell story about the present condition of kidneys. If these are in higher level, they will show that the patient will prone to renal failure in short time. During the T1DM, the Disturbance in Renal functions is caused by high glucose threshold routinely during the course of disease. It is a complication. In the patients the glucose remains high, so it easily filtered through glomerulus of kidneys. So the glucose present in the urine. It will lead to cause disturbance in filtration process in kidneys. And also there is a maximum chance of infections due to the presence of higher glucose level which is used as nutrient. When there is abnormality in renal functions, the urea and creatinine level will be high. All patients showed normal value for urea and creatinine as control patients. But the values of urea specially were little bit at upper limit of the normal range if compared with the normal control. The highest value for urea was 37 mg/dl and the mean value for the urea >27 mg/dl. In comparison the mean value for urea in normal control was 21 mg/dl. There was a very small shift but in normal range and can be controlled by treatment. Creatinine showed normal value in patient as control. The mean value for both control sample and patient sample was almost same (0.8 mg/dl).

The other biochemical is *albumin*. Albumin is the major part of total protein in serum albumin has a number of functions in blood like transportation of metal, vitamins, ions, and other blood component which are water insoluble. It also maintains the oncotic pressure. Its lower value is significant for T1DM. Albumin concentration is seemed to be telling the protein of the body. In general, it is checked in T1DM to see the proteins levels. With losing weight in T1DM, the albumin level will move to fall. All the patients showed normality for albumin level as control. The lowest value among patients was 3.9 g/dl. The mean value for patient was 4.24 g/dl and for control was 4.51 g/dl, which showed that these patients were at normal metabolism for albumin. Another parameter HBA1c (glycosylated hemoglobin), which is most important for T1DM patient to check. HBA1c is hemoglobin fraction that is glycated by plasma glucose level. Hemoglobin is the albumin which is present in the RBC of blood. Hemoglobin is glycated nonenzymatically and this glucose remains binds to hemoglobin HbA1c especially when glucose levels are high in blood. The normal life of RBC is 120 days. It tells that whether glucose level is under controlled for last few weeks or not. On the basis of this value the diabetologist suggest the patient to increase or decrease the insulin dosage. Its concentration measurement is very important for patient health. Among patients, the concentrations of HbA1c were very high, which showed that the glucose levels in patients were not under controlled for last few weeks. In contrast all control samples showed normal values. The mean value was 9.33% which was in group of fair control. The highest value was 14.6, showing poor control of glucose level in blood for last few weeks in a patient.

Insulin concentration measurement also has importance in T1DM disease. Normally the insulin was produced by beta cell of Langerhans of Pancreas. Insulin affects especially the glucose metabolism. It increases the uptake of glucose in to tissue and cells. In these way glucose level decreases in blood and extra‐cellular spaces. Indirectly it also affects the protein and lipid metabolism in body. But in T1DM the insulin level is low or not present. The reason behind this is the destruction of beta cells and production of antibodies against insulin in the disease. For healthy life these patients require exogenous insulin for normal body metabolism. The serum of subjects (patient) contained very low level of insulin. Although some patient contained insulin in normal range, but may be this insulin was that exogenous insulin which were injected. The control individual had normal insulin concentration. The mean values for patients are 2.33 µlU/ml which is very high as compared to control having 5.89 µlU/ml.

Anti‐insulin antibodies are antibodies which are produced in response to insulin. Normally, it does not present or present in very minute quantity. T1DM these anti‐insulin antibodies targeted as foreign particle as shows autoimmunity against its own proteins. As compared to the controls the patient's values were very high. The highest values among patients were 37 U/ml. The mean value for patient is about 14.0 U/ml. But in comparison with the patients, controls have lower range even in the normal range. The mean value for control is 6.0 U/ml. C‐peptide is 31 amino acid containing peptide, which is separated from pro‐insulin when it is change to its active form of hormone insulin. As insulin is not present in T1DM or present in very low amount. So C‐peptide level is in very low amount or not present. So C‐peptide is used to differentiate T1DM from T2DM. The mean value for patient is 0.08 ng/ml, which is negligible if it is compared with controls which have mean value 0.95 ng/ml. So it means that the patient had not any pro‐insulin converted to insulin. Some patient did not have C‐peptide. Controls have c‐peptide in their normal range. The highest value for c‐peptide is 0.4 ng/ml. Leptin is secreted for some metabolic activities especially for lipid metabolism. Leptin for all patient and control individual is in normal range. Leptin can be related to the lipid profile values. As all lipid values are in normal ranges so for that leptin is secreted in normal ranges. It means all patients showed normal lipid metabolism as controls. They were not prone to coronary heart disease.

Next step is to evaluate the molecular aspects of allele HLA‐DQB1. For that two SNPs are focused. The primers DQR as reverse primer and DQL as forward primer were used to check one SNPs and DQAL as forward primer and DQAR as reverse primer were used to check the second SNPs. The annealing temperatures were measured by gradient PCR. Using these primers the all patients genomic DNA were amplified for these SNPs. Only one of all patients shows the homozygous and two of all control show homozygous state for these SNPs. One patient's DNA did not get any amplification for both SNPs. All other patients and controls showed heterozygous state for both SNPs. These results give the idea that these SNPs are moderate protective to cause T1DM.

## 5 CONCLUSION

All these studies show that these patients have normal protein metabolism, normal lipid metabolism but abnormal carbohydrate metabolism in body. These patients have diabetes having no any complication present. But the level of glucose should be maintained in normal ranges. These patients having high titer of high anti‐insulin antibodies respond to clear the insulin level in the body which leads to disturbance in glucose metabolism. As there is no any insulin or its precursor proteins present in the body, so the C‐peptide level at its lowest limit. We selected only two SNPs. There is need to check all 44 haplotype combinations in Pakistan population. For this, there is need of a consortium standard work for T1DM. Also, these patients at their learning age so proper treatments with proper guidance are needed to educate these patients. These patients were on routinely diet prescriptions by physicians.

## AUTHOR CONTRIBUTION

GS, MZ, and MA collected, analyzed the data, and drafted the manuscript. GS and SH designed the study, and directed implementation and data collection. MA provided necessary logistical support. MZ edited the manuscript for intellectual content and provided critical comments on the manuscript.
